# THE RELATIONSHIP BETWEEN MOOD OF CAREGIVERS AND CARE RECIPIENTS WITH DEMENTIA

**DOI:** 10.1093/geroni/igad104.2660

**Published:** 2023-12-21

**Authors:** Yeji Hwang

**Affiliations:** Seoul National University, Seoul, Republic of Korea

## Abstract

Anxiety and depression are common behavioral and psychological symptoms of dementia. Although there are many individual level factors related to their anxiety and depression, it is unknown whether caregivers’ mood influence anxiety and depression of their care recipients with dementia. Therefore, this study was conducted to examine the relationship between anxiety and depression of caregivers and that of care recipients with dementia. This study was a secondary data analysis using the National Health and Aging Trends Study (NHATS) Round 7 data and National Study of Caregiving (NSOC) III. This study used 553 dyads of older adults aged 65 years and older who had probable dementia at the time of the interview and their family caregivers. Anxiety and depression of both the caregivers and care recipients of dementia were measured with the Generalized Anxiety Disorder-2 (GAD-2) and Patient Health Questionnaire-2 (PHQ-2) respectively. Descriptive statistical analyses, chi-square tests, and logistic regression analyses were used. There were significant relationships between the caregivers’ anxiety and the care recipients’ anxiety presentation (Chi2=10.06, p< 0.01). Similar relationships were also found for depression (Chi2=5.22, p=0.02). Logistic regression analyses showed that when caregivers had anxiety or depression, it was more likely that their care recipients show anxiety (OR=2.25, p=0.002, 95% CI=1.35-3.74) or depression (OR=1.78, p=0.023, 95% CI=1.08-2.94). Because dementia caregivers’ negative mood can influence their care recipients’ mood, health care professionals should conduct early interventions for caregivers with negative moods.

